# Microbiological quality of traditional and improved kiln smoked catfish (*Clarias gariepinus*; Pisces; Clariidae) in Lake Chilwa Basin

**DOI:** 10.1002/fsn3.885

**Published:** 2018-11-08

**Authors:** Martin Charles Likongwe, William Kasapila, Mangani Katundu, Placid Mpeketula

**Affiliations:** ^1^ Faculty of Food and Human Sciences Lilongwe University of Agriculture and Natural Resources (LUANAR) Lilongwe Malawi; ^2^ Faculty of Science Chancellor College University of Malawi Zomba Malawi

**Keywords:** *Clarias gariepinus*, *Escherichia coli*, improved smoking kiln, *Salmonella*, smoking, traditional smoking kiln

## Abstract

Microbiological quality of smoked catfish (*Clarias gariepinus*) locally known as Mlamba was assessed in this study where traditional and improved smoking kilns were used to smoke fish. Catfish is common fish caught in abundance in the Lake Chilwa basin, and the fish is usually smoked to reduce postharvest losses and increase shelf life. Samples were collected in sterile polythene bags, well labeled, and collected in cooler boxes transported ready for laboratory analysis. One gram representative sample was obtained aseptically from the muscle of the fresh and smoked catfish (Mlamba) samples. The samples were grounded, and fourfold serial dilutions (10^−1^–10^−4^) of the homogenized samples were made using sterile distilled water. Fish samples were analyzed for total plate count (TPC), *Escherichia coli* counts, and pathogenic organisms (*Salmonella*) following the methods prescribed by AOAC (Official methods of analysis, Association of Official Analytical Chemistry, Arlington, VA, 2000). Each analysis was carried out in triplicates. There were significant differences (*p* = 0.05), with respect to total viable bacterial counts between traditional kiln smoked and improved kiln smoked catfish (5.6 × 10^6 ^cfu/g, 1.9 × 10^6 ^cfu/g, respectively). Traditional kiln smoked catfish harbored significantly higher total viable counts as well as a higher population of *E. coli* compared to improved kiln smoked catfish. However, for both types of smoking kilns there were detected levels of pathogenic bacteria *Salmonella* with traditional kiln smoked catfish containing 2.1 × 10^4^ cfu/g which were significantly higher than amount found in improved kiln smoked catfish (1.5 × 10^4 ^cfu/g; *p* = 0.05). *Salmonella* is a microbe of public health importance and has implications on the handling and source of the fish.

## 1 BACKGROUND

Fisheries and aquaculture sector has been pivotal to millions of people around the world in providing a source of income and livelihood. According to FAO (2014), it is estimated that 58.3 million people were involved in the primary sector of capture fisheries and aquaculture in 2012.

According to the Government of Malawi (2012), fishing is significant to Malawi as it contributes to the livelihood of millions of Malawians especially those from rural Malawi and economic growth of the country. The sector contributes about 4% to the Gross Domestic Product (GDP) for Malawi and employs about 60,000 fishers and indirectly employ over half a million Malawians through processing, fish marketing, and boat building and repair. Many of these employees are rural women involved in fish processing and marketing. This means that fishing has great impact on the livelihood of Malawians and plays a pivotal role to the economy of Malawi as a decline in the fisheries will have a great impact on the livelihood of Malawians.

Considerable quantity and quality losses occur in fish after being caught because fish is a highly perishable commodity. It has been recorded that no other food provides so much observed evidence of serious loss from harvest to consumption and so little documentation of the overall proportion of losses from fish production (Aberoumand, 2013; ECA, 1984). Fish after being caught is very susceptible to deterioration if not preserved and processed (Okonta & Ekelemu, 2005). The deterioration that sets in when fish dies is both physiological and microbial; these invariably degrade the quality of fish (Eyo, 2001). According to Daniel, Ugwueze, and Igbegu (2013), bacterial growth and invasion of fish are prohibited by the body's natural defense system when fish is alive, but after death, the defense system breaks down and the bacteria grow and invade the flesh. Chemical breakdown of protein content, fat content (agent of rancidity and off‐flavor), and the water content/water activity contributes to quick spoilage of fish. These physiological and microbial activities make the fish unfit for human consumption within about 1 day of capture, unless it is subjected to some form of processing or preservation.

Postharvest losses of fish take place in the fish value chain in various degrees or extent. According to Signa (2014) postharvest losses (PHL) can be defined as the decrease in quantity, or quality or monetary value of fish in the supply or value chain. The losses will generally result in the loss of income to the people involved in the value chain and also loss in the availability of fish as food; hence, they represent a major food security concern in Africa, where many people are food insecure. In Malawi, about 30% of fish caught in Malawi is lost through postharvest losses due to poor processing, packaging, and transportation (Jamu, Botha, & Luhanga, 2012). According to Mutungi and Affognon (2013), in Malawi almost 90% of the fish from capture fisheries is preserved before being sold. Smoking accounts for 40% and sun‐drying takes up 50% of the fish preservation methods, whereas only 10% is handled and sold as fresh, chilled, and frozen. There are a number of processing facilities that are used which include the traditional type, like dug‐out smoking ovens and drying racks made of reeds and mats; improved facilities have also been adopted in some areas such as Bena kiln (improved Ivory Coast kiln) and wire drying racks.

*Clarias gariepinus* (catfish) which is locally known as Mlamba is a common fish species that is landed in abundance in Lake Chilwa. The fish is commonly smoked by fish processors to preserve and reduced postharvest losses. Smoking also reduces moisture content of the fish which is a determinant of the quality in dried food products and that a moisture content of 15% or less could inhibit microbial growth in dried fish (Salaudeen & Osibona, 2018). The smoking is done using traditionally built smoking kilns which require a lot of firewood, time, and labor to smoke the fish. In order to reduce problems associated with traditional smoking kilns and reduce postharvest, Chancellor college in collaboration with WorldFish with funding from International Development Research Centre (IDRC) introduced improved smoking kilns which use lesser firewood in the Lake Chilwa basin for use in processing fish. This study therefore was conducted to assess the effect of using traditional and improved smoking kilns on the microbiological quality of smoked catfish in the Lake Chilwa basin.

## 2 MATERIALS AND METHODS

### 2.1 Study sites

The study was carried out in Zomba District at Mchenga beach in the Lake Chilwa basin where a CultiAf project funded by International Development Research Centre (IDRC) and Australian Centre for International Agriculture Research (ACIAR) was being implemented.

### 2.2 Fish processing methods

**Figure 1:**
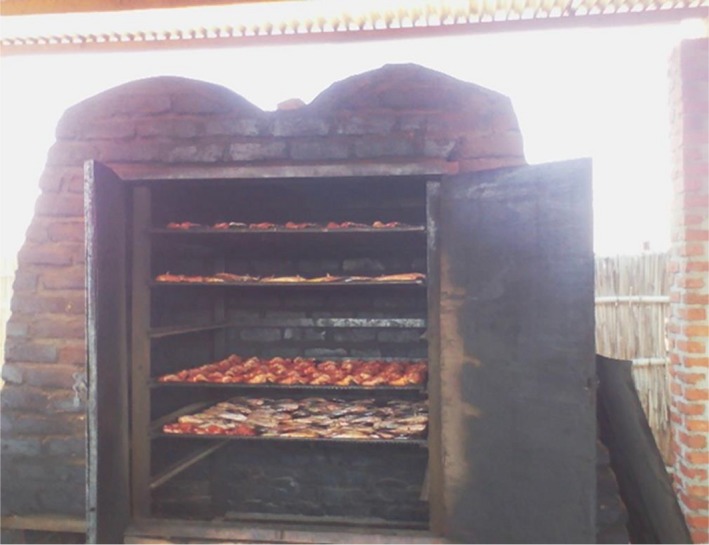
Improved smoking kiln with doors open with catfish being smoked

**Figure 2:**
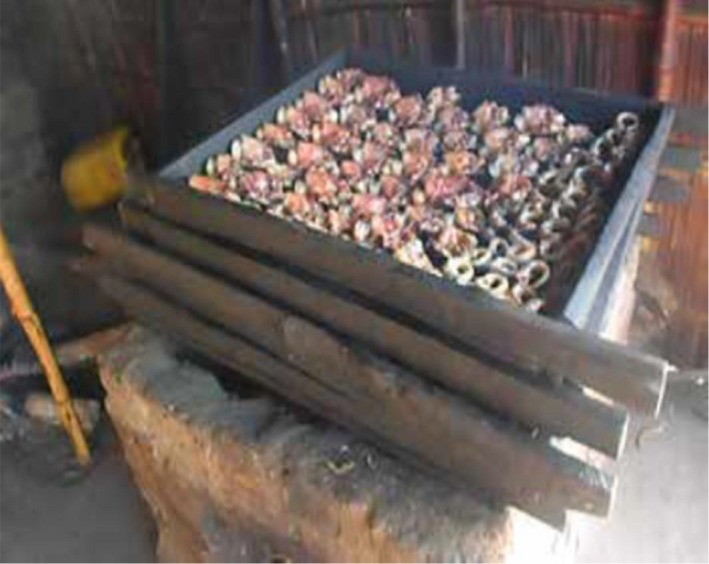
Traditional smoking kiln with catfish being smoked

**Figure 3:**
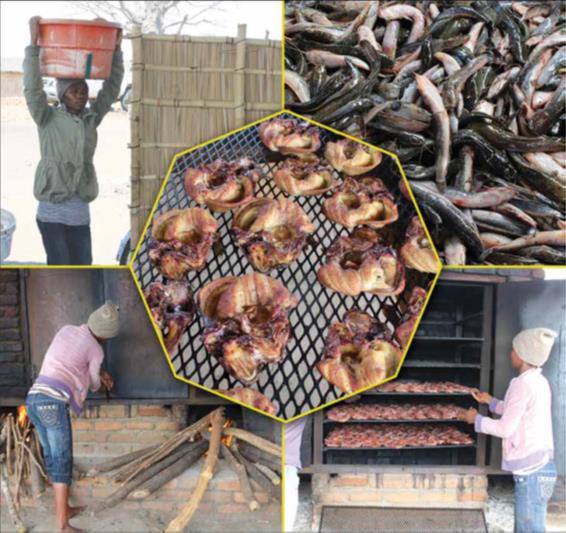
A fish processor in Lake Chilwa Basin showing the fish smoking process using improved smoking kiln

*Clarias gariepinus* fish species were collected from fish trawlers at Mchenga beach (landing site). The fish were thoroughly washed, split open, and eviscerated. The fish were then put on smoking trays and placed in smoking kilns for smoking. Smoking of the fish was done using improved smoking kilns (Figure 1) and traditional smoking kilns (Figure 2). Improved smoking kilns have six trays, and during smoking, the doors of the kiln are closed which enhances smoking as smoke is not lost since it is in an enclosure (Figure 3). In traditional smoking kilns, the number of trays for smoking varies from 4 to 6 depending on size of kiln.

### 2.3 Study design

A completely randomized design was used when samples were collected from fish processors after processing for microbial analysis. All the samples were collected from fish processors soon after processing, a total of 50 samples for each processing method. The samples were put in new polythene bags, labeled, and collected in cooler boxes transported ready for laboratory analysis. Fresh samples were immediately put on ice before being taken to the laboratory, while other samples were processed and collected immediately after and transported to the laboratory. Both the fresh and processed samples were collected from the same batch after purchase. At the laboratory, samples were composited from each batch for subsequent analysis. Fresh fish samples were used to determine the level of contamination the fish had before processing.

### 2.4 Moisture content determination

New aluminum dishes free from any contaminants were used for weighing samples. The dishes were dried for 1 hr at a temperature of 100 ± 5°C to dry. After drying, the dishes were placed in a desiccator, using a tong, for 30 min to cool; after which they were weighed using an analytical balance with 0.1 g sensitivity following methods of AOAC (2010) and Bradley (2010). The samples were then weighed (2 g for each sample) and placed in an oven at 100 ± 5°C for 5 hr (Bradley, 2010). Samples were removed after 5 hr from the oven and placed in a desiccator, using a tong for 30 min to cool after which they were weighed using the same analytical balance. The heating and weighing procedure was repeated after every 30 min until a constant weight was reached.

Calculation of percent (%) moisture content was done using the formula;

$$\text{Percent moisture content}=\left(\left(W_{1}-W_{2}\right)\times 100\right)/W_{1}$$

Where:

$$W_{1}=\text{Initial weight of sample}$$

$$W_{2}=\text{Weight of sample after drying and reaching constant weight}.$$

### 2.5 Microbiological analyses

One gram representative sample was obtained aseptically from the muscle of each of the smoked and dried samples. The samples were grounded, and fourfold serial dilutions (10^−1^–10^−4^) of the homogenized samples were made using sterile distilled water. Each analysis was carried out in triplicates. The fish samples were analyzed for total plate count, total coliforms, *E. coli*, and pathogenic microorganisms (*Salmonella*). All microbial analyses were done following the methods prescribed by AOAC (2000). The results were reported in colony‐forming units per gram (cfu/g).

Aliquots of suitable dilutions were transferred separately to plates count agar for total plate count, *Salmonella–Shigella* agar selective media for isolation of *Salmonella*, MacConkey agar selective media for *E. coli* count, and Violet Red Bile agar selective media for total coliforms count. Morphological characteristics were used to identify the different bacteria in the agar during plate count. As *Salmonella* does not ferment lactose, but produce hydrogen sulfide (H_2_S) gas, the colonies observed were colorless with black centers.

### 2.6 Data analysis

All analyses were done using the SPSS software for Windows (version 16; SPSS Inc., 2006, Chicago, IL, USA). One‐way analysis of variance (ANOVA) where *p* < 0.05 was applied to the different sample values obtained.

## 3 RESULTS

The study showed that the moisture content of improved kiln and traditional kiln smoked fish was not significantly different (33.51 ± 0.56% and 30.15 ± 3.60%) as shown in Table 1. Smoking temperatures were higher in improved smoking kilns than in traditional smoking kilns (138.5–177°C and 113.7–158.5°C, respectively) as shown in Table 1. There were recorded lower microbial counts of total plate counts, *Salmonella*, Total coliforms, and *Escherichia coli* in fish smoked in improved smoking kiln (1.936 × 10^6^, 1.550 × 10^4^, 2.00 × 10^4^ and 0 cfu/g) than fish smoked in traditional smoking kilns (5.656 × 10^6^, 2.100 × 10^4^, 4.53 × 10^4^ and 6.67 × 10^2 ^cfu/g) as shown in Table 2.

**Table 1:** Moisture content of smoked catfish and smoking temperature

	Moisture %	Temperature °C
*Clarias* (Mlamba) fresh	77.44^a ^± 0.30	
*Clarias* (Mlamba) traditional kiln smoked	30.15^b ^± 3.60	113.7–158.5
*Clarias* improved kiln smoked	33.51^b^ ± 0.56	138.5–177

The superscript shows there is significant differences in the moisture content in the fish processed differently. The two smoking kilns did not have significant differences in moisture content.

**Table 2:** Mean microbial counts in colony‐forming units per gram (cfu/g) in smoked Catfish (*Clarias gariepinus)*

Sample id	Total plate counts	Salmonella	Total coliforms	*Escherichia coli*
Fresh *Clarias*	1.344 × 10^6^	3.575 × 10^4^	1.80 × 10^5^	4.00 × 10^4^
Traditional kiln whole smoked *Clarias*	5.656 × 10^6^	2.100 × 10^4^	4.53 × 10^4^	6.67 × 10^2^
Improved kiln whole smoked *Clarias*	1.936 × 10^6^	1.550 × 10^4^	2.00 × 10^4^	0

## 4 DISCUSSION

The moisture content of smoked fish in this study was higher than the allowable limit of 6%–8% as recorded by Olayemi, Raji, and Adedayo (2012) and much higher than those reported by Salaudeen and Osibona (2018) who found moisture content of 15.84% and 12.70% in traditional kiln and improved kiln smoked catfish, respectively. The smoked fish was collected from fish processors in the way they smoke the fish. The study shows that fish processors do not smoke they fish to the recommended moisture content that would enhance the shelf life of the smoked fish as they usually sell or take the fish to the market immediately after smoking. There were no significant differences in the moisture content of the traditional kiln and improved kiln smoked fish. The moisture content has an effect on the water activity of the smoked fish which influences microbial growth on the smoked fish.

The highest mean total viable count of 5.656 × 10^6^ was found in traditional kiln smoked *C. gariepinus*. The study has shown that traditional kiln smoked *C. gariepinus* had the higher microbial counts registered than the improved kiln smoked *C. gariepinus* as shown in Table 2. The study has shown that smoking reduces the amount of *Salmonella*, total coliforms, and *E. coli* found in the *C. gariepinus* fish as highest figures were found in fresh samples 3.575 × 10^4^, 1.80 × 10^5^, and 4.00 × 10^4 ^cfu/g, respectively. In improved kiln smoked fish, the microbial load was lower than in the traditional kiln smoked and fresh fish meaning that higher temperatures during smoking resulted in more microorganisms being killed and not able to multiply. In improved kiln smoking, fish is also hygienically handled and fish is in an enclosure which reduces infestation of microbes which is in agreement with Immaculate, Sinduja, and Jamila (2012).

Though total coliforms were found in the improved kiln smoked samples as shown in Table 2, it was noted however that these samples had lower amounts of coliforms and *E. coli* found in them (2.00 × 10^4^ and 0 cfu/g) respectively as compared to traditional kiln smoked samples (4.53 × 10^4^ and 6.67 × 10^2 ^cfu/g). In their study, Salaudeen and Osibona (2018) found that total bacteria viable counts were high in fresh fish and reduced in smoked catfish which was true in this study especially for Salmonella, *E. coli*, and total coliforms count. Adegunwa, Adebowale, Olisa, and Bakare (2013) previously reported that the International Commission on Microbiological Specification for Food (ICMSF) has set the maximum recommended bacteria count for good quality fish products at 5.0 × 10^5^ cfu/g and 1 × 10^7 ^cfu/g as the maximum for marginally acceptable quality products, and for *Listeria monocytogenes* and *Salmonella* spp., the level in the presence of organism is zero tolerance. In research, the findings are within the acceptable level of bacteria count except for *Salmonella* species levels that were observed in almost all samples. The results may be attributed to handling practices during smoking by the fish processors as the fish is handled with hands during and after processing which may introduce bacteria on the fish after processing. Smoking of the fish was also at variable temperatures because of the amount of fire available during the smoking period which meant that microorganisms were not exposed to the same amount of heat that would kill them completely. Abolagba and Iyeru (1998) and Daniel et al. (2013) have reported that varying microbial load in smoked fish would result from lack of proper smoking and hygienic handling of the smoked fish.

It has been shown in this research as shown in Table 1 that moisture content of the smoked fish was high which would still encourage microbial growth despite the amount of microorganisms being reduced during smoking. It was observed during the research that the smoked fish were spread in the open on wire mesh to cool after smoking which was exposing the fish to the environment which was somehow humid and this may have encouraged the smoked fish to increase in moisture by absorbing it from the environment. This increase in moisture may have increased activity of microorganisms. Eyo (2001) stated that smoked fish samples may have a relatively high water activity which would encourage microbial growth. Akinwumi and Adegbehingbe (2015) have recorded that smoking at high temperatures has potential to control fish microbial contamination, although the heat used may not suffice to kill all the microbial contaminants. Temperatures as high as >600°C when used in smoking would result in inactivating vegetative microorganisms, but this may bring in concerns on the sensory quality of the smoked fish.

## 5 CONCLUSION AND RECOMMENDATIONS

The study findings have shown that improved smoking kilns are more effective in reducing microbial load of the processed fish and that more fish are processed per unit time as compared to traditional smoking kilns. Since the moisture contents of the smoked fish were still high, it is recommended that the fish should be smoked further to lower moisture content which would result in lower microbial load and increased shelf life of the smoked fish.

## CONFLICT OF INTEREST

The authors declare that they have no conflict of interest.

## ETHICAL REVIEW

The study did not involve any human and animal testing of the samples. The research protocol was approved and given ethical clearance by National Commission for Science and Technology in Malawi (protocol approval number: P.05/16/101).

## INFORMED CONSENT

Written informed consents were obtained from all study participants.
